# Self-assembled kanamycin antibiotic-inorganic microflowers and their application as a photocatalyst for the removal of organic dyes

**DOI:** 10.1038/s41598-019-57044-z

**Published:** 2020-01-13

**Authors:** Ratan W. Jadhav, Duong Duc La, Vishal G. More, Hoang Tung Vo, Duy Anh Nguyen, Dai Lam Tran, Sheshanath V. Bhosale

**Affiliations:** 1 School of Chemical Sciences, Goa University, Taleigao Plateau, Goa 403 206 India; 2 Institute of Chemistry and Materials, Hanoi, Vietnam; 3 Environmental Institute, Vietnam Maritime University, Haiphong city, Vietnam; 4 Institute of Tropical Engineering, Vietnam Academy of Science and Technology, Hanoi, Vietnam

**Keywords:** Self-assembly, Photocatalysis

## Abstract

Construction of hybrid three-dimensional (3D) hierarchical nanostructures via self-assembly of organic and inorganic compounds have recently attracted immense interest from scientists due to their unique properties and promise in a large range of applications. In this article, hybrid flower structures were successfully constructed by self-assembly an antibiotic, kanamycin, with Cu^2+^. The flower-like morphology was observed by scanning electron microscopy, to be approximately 4 µm in diameter and about 10 nm in thickness. FTIR spectroscopy and X-ray diffraction confirmed the antibiotic-inorganic hybrid structure was uniform composition, and showed crystallinity due to ordered self-assembly. The hybrid flowers showed high photocatalytic activity towards degradation of methyl blue during 240 minutes under visible light irradiation. A possible mechanism of photocatalytic activity was also proposed, that exposes the inherent advantages in using antibiotic-inorganic hybrid flowers as photocatalysts, where self-assembly can be used to generate active, high surface area structures for photodegradation of pollutants.

## Introduction

Recently, intensive attention has been focused on methods to tailor the morphology of nanostructured materials such as core/shell nanoparticles, nanoplates, nanowires, nanofibers, nanotubes and nanoflowers^1–5^. With the extensive possibility of applications in gas sensing, photocatalysis, energy storage, adsorption, chemical and biological sensing, separation, flower-like structures have been intensively studied in recent years^6,7^ Three dimensional flower-like morphologies in particular can be constructed from various materials such as metal oxides, hydroxides^8^, carbon-based materials^5,9,10^, and other materials^11–13^. These flower-like structures have a high ratio of surface to volume, which can be effectively utilized in many applications including, but not limited to, chemical and biological sensing, catalysis, and adsorption^14–17^. Even though many approaches have been employed to fabricate three-dimensional flower-like structures, conventional synthetic methods are complicated, involving strictly controlled conditions such as inert media, high temperature, use of toxic chemicals or high pressure. Thus, finding a simple and cost-effective approach for the synthesis of three-dimensional flower-like structures is worthy of further research.

In the field of advanced nanomaterial fabrication, self-assembly has attracted considerable attention due to the possible formation of dynamic and complex systems from basic building blocks, and may afford materials for applications in areas such as sensors, energy storage, and optics^18–22^. Among the classes of self-assembled nanostructures, organic and inorganic compounds can be spontaneously integrated as different components via hierarchical self-assembly to obtain hybrid materials^23–25^. In particular, several three-dimensional functional hybrid flower-like structures had been created via self-assembly^24,26–28^. Ge *et al*. used the inorganic component of copper (II) ions with various proteins as the organic component to fabricate hybrid organic–inorganic nanoflowers^29^. The main driving force for formation of flower-like morphology was the interaction between the protein and copper ion, and the resultant hybrid flower showed enhanced enzymatic stability and activity compared to that of the free enzyme. In another study, 3D nanoflowers were successfully fabricate by using copper(II) ions as the inorganic component self-assembled with various biosurfactants as the organic component^30^. The mechanism of growth of nanoflower in this instance was complex formation between the biosurfactant molecules and the copper ions, and these complexes then became nucleation sites for primary crystals of copper phosphate, which indicate that the interaction between biosurfactant and copper ions leads to the petal formation, shaping into three-dimensional nanoflowers. These organic-inorganic hybrid nanoflowers were of high stability and displayed catalytic activity for the degradation of cationic dyes.

Antibiotics of the aminoglycoside type (e.g., tobramycin, kanamycin, neomycin B) are formed by the link between two amino sugar molecules with an aminocyclohexanol unit by glycosidic bonds. The amine groups in antibiotics can form molecular complexes with various metal ions or inorganic compounds. Inspired from self-assembly in nature, herein we propose a facile approach to fabricate antibiotic-inorganic hybrid flowers via self-assembly, using copper ions as inorganic component and kanamycin (an antiobiotic) as the organic component. The complexation of copper ions with kanamycin are responsible for nucleation and growth of the flower-like structures. The photocatalytic behavior of the three dimensional inorganic-organic hybrid materials was investigated for the first time along with a brief explanation of the possible photocatalytic mechanism.

## Results and Discussion

**Figure 1:**
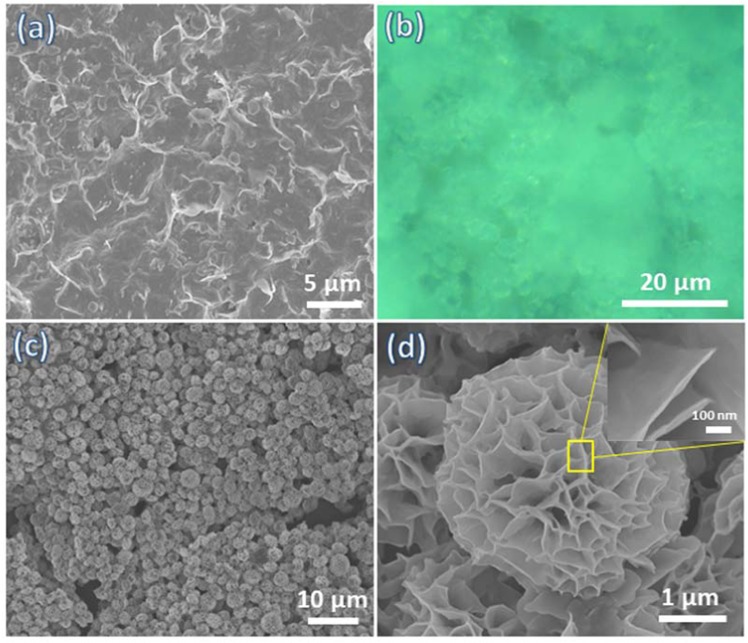
SEM images of solids obtained from (**a**) Cu_3_(PO_4_)_2_.3H_2_O crystal, (**b**) optical image and (**c**, **d**) SEM images of kanamycin-Cu_3_(PO_4_)_2_ hybrid flowers.

The hybrid flower-like structures were fabricated by adding Kanamycin molecules, one of the most commonly used antibiotics, into the aqueous CuSO_4_ solution in. the presence of phosphate buffer saline (PBS). Figure 1a and S1 reveals the morphology obtained and isolated after CuSO_4_ was present in PBS buffer following the procedure outlined in the experimental section, it is observed that no structures are formed without the addition of Kanamycin. When a certain amount of Kanamycin was introduced into the PBS-containing CuSO_4_ solution, green precipitates were observed after 1 day. These green precipitates had uniform flower structures with an average diameter of 4 µm and a petal thickness of less than 10 nm (Figure b,c & d). This flower-like morphology has high surface to volume ratio, and the structures observed showed good monodispersity. The effect of Kanamycin concentration on the morphology of the hybrid structures is shown in Fig. S2. At a low concentration of 20 µl Kanamycin (120 mM), a uniform flower-like morphology is observed as illustrated in Fig. S2a. A further increase of Kanamycin concentration leads to the flower structures starting to aggregate at Kanamycin additions of 40 and 60 µl (Figure S2b,c) and finally microsheet structures are observed with Kanamycin additions of 80 and 100 µl (Fig. 1Sd&e).

**Figure 2:**
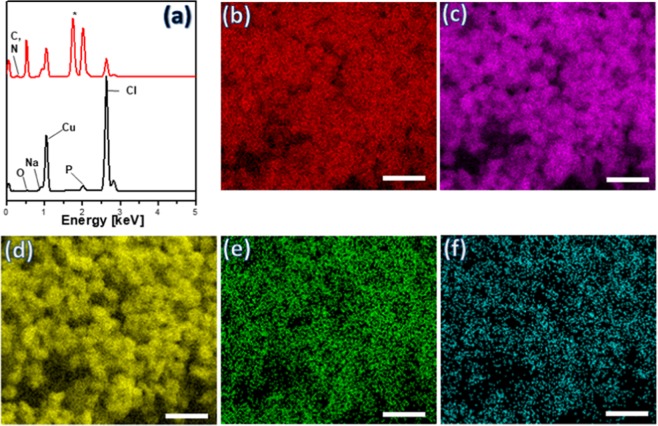
(**a**) EDS spectra of Cu_3_(PO_4_)_2_ crystals (black line) and kanamyci-Cu_3_(PO_4_)_2_ hybrid flowers (red line), with the EDS element mapping of (**b**) Cu, (**c**) P, (**d**) O, (**e**) C, (**f**) N with scale bar of 10 µm.

Energy Dispersive Spectroscopy (EDS) was utilized to confirm the elemental composition of the isolated structures. Figure 2a shows the EDS patterns of the precipitates before and after addition of kanamycin, with the appearances of C and N peaks confirming the formation of kanamycin-inorganic hybrid structures in the final product. Furthermore, the elemental mapping (Fig. 2b–f) demonstrates that C, N, Cu, P and O elements are homogeneously distributed throughout the hybrid flower. This may be attributed to complex formation between kanamycin and Cu_3_(PO_4_)_2_.3H_2_O^30^.

**Figure 3:**
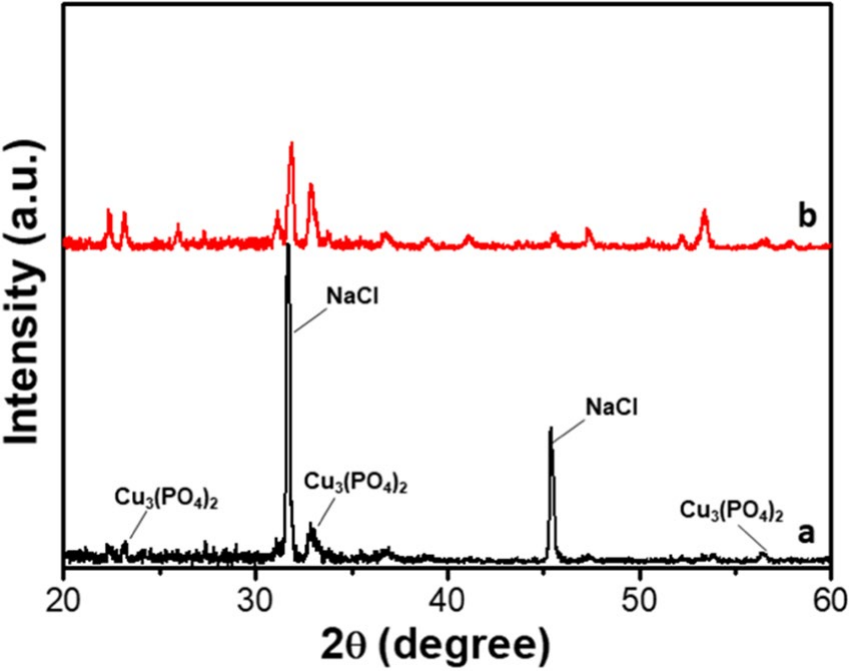
XRD patterns of Cu_3_(PO_4_)_2_ crystals (black line) and kanamycin-Cu_3_(PO_4_)_2_ hybrid flowers (red line).

The crystallinity of the kanamycin-inorganic hybrid flowers was investigated by collection of XRD patterns (Fig. 3 & S3). It is clear shown in Fig. S3 that the Kanamycin monomer is amorphous in nature. The peaks that appeared in the XRD pattern of the sample without kanamycin (black line in Fig. 3) confirms the presence of Cu_3_(PO_4_)_2_ and NaCl crystals. In the XRD pattern of the hybrid flowers (red line), it can be seen that besides the existence of peaks for Cu_3_(PO_4_)_2_ and NaCl, extra diffraction peaks are observed, that confirms the existence of ordered structures in the kanamycin-inorganic hybrid flowers, indicating that directed growth through non-covalent interactions has led to crystallinity in the flowers.

**Figure 4:**
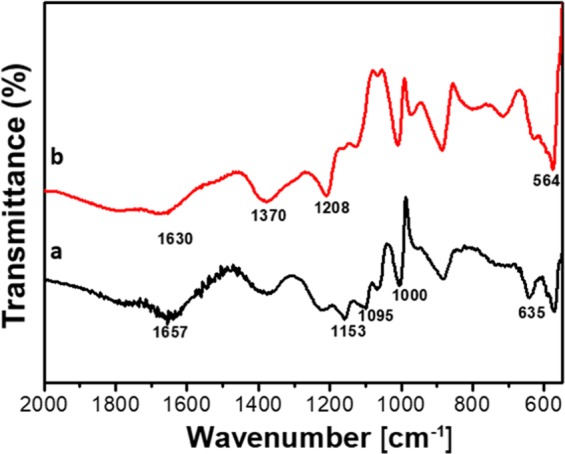
FTIR spectra of Cu_3_(PO_4_)_2_ crystals (black line) and kanamycin-Cu_3_(PO_4_)_2_ hybrid flowers (red line).

The formation of the kanamycin-inorganic hybrid flowers was also studied by FTIR spectroscopy. Figure 4 shows the FTIR spectra of Cu_3_(PO_4_)_2_ and kana-Cu_3_(PO_4_)_2_ hybrid flowers. In the FTIR spectrum of Cu_3_(PO_4_)_2_ (black line), the broad sketching at 1657 cm^−1^ is attributed to adsorbed water. The characteristic peaks at 1153, 1095 and 1000 cm^−1^ are assigned to the bending vibrations of Cu–OH (asymmetric), and symmetric O = P and P-O stretching vibrations, respectively^27,31^. The characteristic bands at 564 and 635 cm^−1^ are assigned to the O = P-O bridging phosphorous, and confirm the presence of phosphate groups^32^. In the FTIR spectrum of the kanamycin-Cu_3_(PO_4_)_2_ hybrid flowers (red line), while the characteristic peaks at 1153, 1095, 1000, 635 and 564 cm^−1^ demonstrate the presence of the Cu_3_(PO_4_)_2_, the bending vibration at 1208 cm^−1^ can be assigned to phenyl ring-carbon sketching^33^, which further confirms the presence of kanamycin molecules within the hybrid flowers. Furthermore, no obvious new characteristic peaks are observed in the FTIR spectrum of kanamycin-Cu_3_(PO_4_)_2_ hybrid flowers, confirming the self-assembly pathway to form the flower structures, instead of covalent bonding.

**Figure 5:**
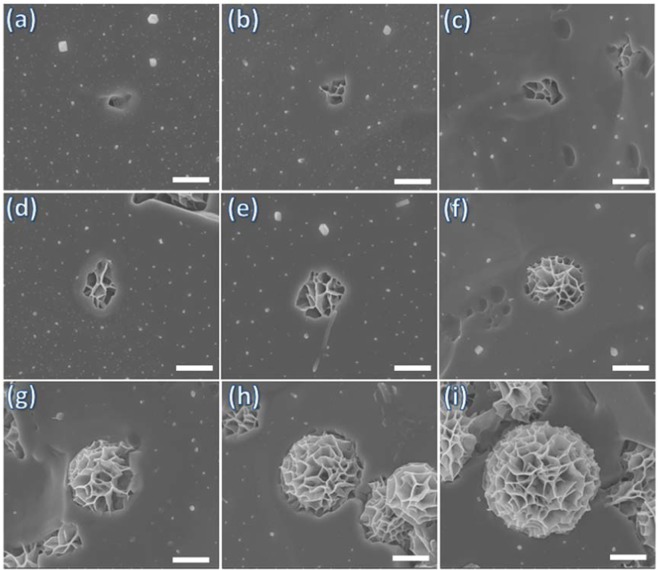
Step-wise nucleation and growth mechanism of the Kanamycin- Cu_3_(PO_4_)_2_ hybrid flowers with time interval of 1 hour for each step-wise growth. Scale bar is 1 µm.

Figure 5 illustrates the step-wise growth of kanamycin-Cu_3_(PO_4_)_2_ hybrid flowers upon addition of Kanamycin into the PBS-buffered Cu^2+^ solution at various stages of flower growth. It can be observed that the hybrid flowers initially nucleate on a small site, and then gradually grow into the full flower-like structures via directed growth of the petals. It is a reasonable assumption that the coordination between the antibiotic and Cu^2+^ is the main driving force for the formation of hybrid flowers. The amine groups in the kanamycin compounds can complex with Cu^2+^, leading to growth of the petals. At the beginning, the amine moieties are diluted in aqueous media to form separate sites on the surface of kanamycin backbone. Then these amine moieties coordinate with the Cu^2+^ binding sites of the copper phosphate to form the separate petals, which eventually shape into full hybrid flowers.

**Figure 6:**
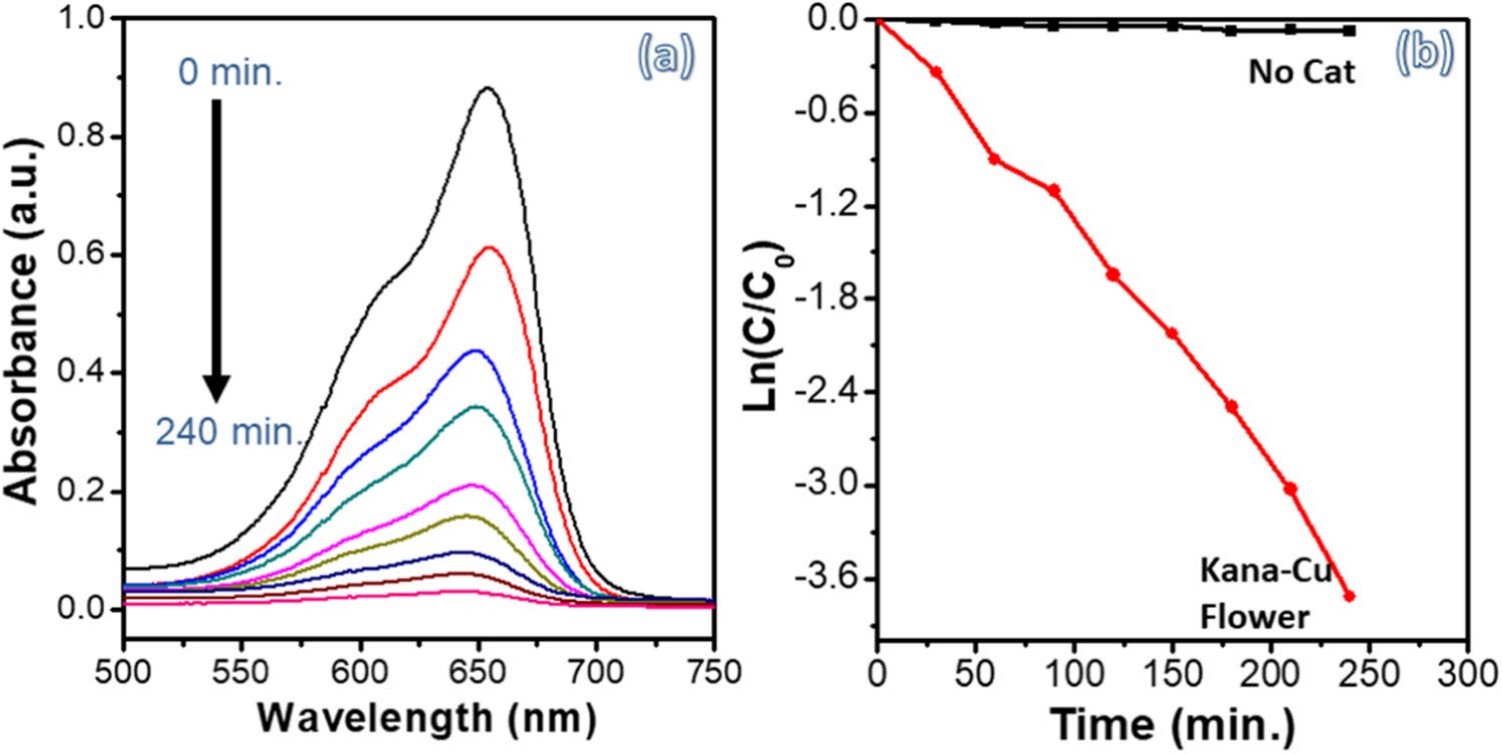
(**a**) Photocatalytic performance and (**b**) kinetic simulation curve for methyl blue by Kanamycin- Cu_3_(PO_4_)_2_ hybrid flowers for MB degradation.

Copper phosphate-based materials have been previously shown to have good photocatalytic properties^34,35^, due to their broad visible light absorption range. In this work, the photocatalytic behavior of the kanamycin- Cu_3_(PO_4_)_2_ hybrid flowers for the degradation of methyl blue (MB) was studied as a model reaction, and results are shown in Fig. 6 and Figure S5. The decrease in the absorption peak at 653 nm as a function of time due to MB degradation was employed to assess the photocatalytic activity. Illustrated in Figure S5 is the C/C_o_ versus time plot of MB under various photocatalytic conditions, where C_o_ is the initial concentration of the dye and C is the concentration at time t. The absorption spectrum of MB solution and kanamycin-Cu_3_(PO4)_2_ hybrid flowers in water are illustrated in Figure S4 indicating that the hybrid flowers show no dissolution in the water. Figure 6a is the absorption spectrum of the mixed solution of MB and kanamycin-Cu_3_(PO4)_2_ hybrid flowers after exposed to the light source with different time. It is apparent from Fig. 6a and S5 that the hybrid flowers display excellent photocatalytic performance for the degradation of MB, as after 240 minutes of irradiation time under visible light, virtually 100% of the MB has degraded. No degradation of the MB dye was observed without using the photocatalyst, which suggests that there is no self-sensitized degradation of MB occurring under these conditions. The kinetics of the photocatalytic reaction of kanamycin- Cu_3_(PO_4_)_2_ hybrid flowers for the degradation of MB were investigated through the plot of ln *(A*_*t*_*/A*_0_) vs. time, where *A*_*o*_ is the intensity at time zero, and *A*_*t*_ is the peak intensity at time t (Fig. 6b). It can be calculated from the plot that the degradation rate constant of the MB by kanamycin- Cu_3_(PO_4_)_2_ hybrid flowers is 15 × 10^−3^ min^−1^, which is comparative with other reported photocatalysts^36–38^.

**Figure 7:**
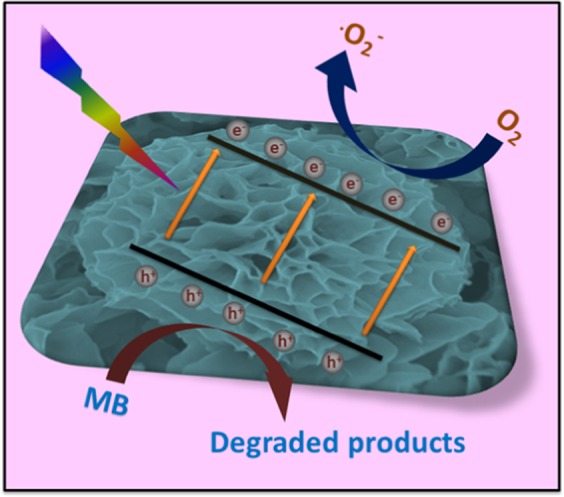
Possible photocatalytic mechanism of Kanamycin- Cu_3_(PO_4_)_2_ hybrid flowers as photocatalyst toward methyl blue dye.

It is well known that copper phosphate nanomaterials display good photocatalytic performance under visible light irradiation^34,35^. With incorporation of kanamycin with copper phosphate, flower-like materials with a higher surface area have been fabricated, which is responsible for enhanced photocatalytic performance. The complexation of the kanamycin molecule with copper phosphate may also broaden the light absorbing region, as well as increase the charge separation ability of the resultant photocatalyst to enhance the photocatalytic activity. Based on the literature and this concept, we propose a possible photocatalytic mechanism of the kanamycin-copper phosphate hybrid flowers for the degradation of organic dyes as shown in Fig. 7. Under visible light irradiation, the kanamycin-copper phosphate hybrid flowers generate electrons/holes pairs by the moving of electrons through bandgap energy from the valence band to the conduction band of the hybrid flowers^39^. The generated holes move to the surface of the petals to react with H_2_O or OH^−^ to form active species such as.OH, and these species will reduce the dye molecules to less harmful degraded products^40^. On the other side of the reaction, the generated electrons will oxidize oxygen in water to form O_3_^−^ radicals^37,41^.

In summary, the growth mechanism from nucleation to a full flower structure was studied by SEM studies, indicating that the coordination between amine moieties in kanamycin and Cu^2+^ was responsible for the nucleation and growth of flower petals, eventually leading to a full flower structure. We have successfully fabricated an organic-inorganic hybrid flower-like structure by complexation between a common antibiotic (kanamycin) and Cu^2+^ in PBS solution. The resultant kanamycin- Cu_3_(PO_4_)_2_ complex has a uniform flower-like structure with an average diameter of 4 µm and a petal thickness of less than 10 nm. The coordination between Cu^2+^ ions and amine groups in the kanamycin is the main driving force for formation of the flower-like morphology. The kanamycin- Cu_3_(PO_4_)_2_ hybrid flower revealed remarkable photocatalytic activity for the removal of methylene blue under simulated sunlight irradiation, with the degradation rate constant of 15 × 10^−3^ min^−1^, which is comparative with reported photocatalysts. The kanamycin- Cu_3_(PO_4_)_2_ hybrid flower can be considered as a modified semiconductor material, which can absorb the light energy in visible region to generate e/h pairs for the degradation of methyl blue dye. With such a high photocatalytic performance, the organic-inorganic supramolecular flowers are promising photocatalyst for environmental treatment of contaminated wastewaters, as well as a contribution that provides insight into the construction of three-dimensional (3D) hierarchical nanostructures. We are currently working on self-assembly of kanamycin and other antibiotics with various metals, which may lead to a deeper understanding of the underlying mechanism that leads to the excellent photocatalytic performance of this antibiotic-inorganic hybrid flower.

## Experimental Section

### Materials and methods

Kanamycin was obtained from TCI, Chennai, India. Copper sulfate (Cu_2_SO_4_), phosphoric acid (H_3_PO_4_, sodium chloride (NaCl), potassium chloride (KCl), methyl blue (MB) and ethanol were obtained from Ajax Finechem (Australia). All chemicals were utilized without any further purification. The nanostructured morphology, composition, as well as elemental distribution in kana-Cu_3_(PO_4_)_2_ hybrid flowers were studied by scanning electron microscopy (SEM), Energy Dispersive spectroscopy (EDS) and EDS mapping using an EDS-integrated FEI Nova NanoSEM (Hillsboro, USA, operating under HV and Stage bias condition of 15 KeV, samples were coated with Pt) and an Everhart Thornley Detector (ETD). Ultraviolet-visible (UV-Vis) absorption measurements of samples in solution were collected using a Cary 50 Bio spectrophotometer with a cell of 1 cm path length. A BrukerAXS D8 Discover instrument with a general area detector diffraction system (GADDS) using a Cu Kα source was utilized to obtain XRD patterns of the hybrid flowers. Fourier transform infrared (FTIR) measurements were performed on a PerkinElmer D100 spectrometer in attenuated total reflectance mode.

### Synthesis of kanymicin-Cu_3_(PO_4_)_2_ hybrid flowers

In the typical procedure for the synthesis of the hybrid flower-like structures, various concentrations of kanamycin were prepared by diluting kanamycin in 3 ml of phosphate buffer saline (PBS, pH 7.4). Then, 20 ml of aqueous Cu^2+^ solution (120 mM) was gradually introduced into the kanamycin solution. The mixed solution was incubated for 1 day under ambient conditions. The precipitate was filtered, rinsed and dried thoroughly, and kept in the dark for further characterization.

### Photocatalytic investigation

The photocatalytic performance of the kana-Cu_3_(PO_4_)_2_ hybrid flowers was investigated for the methyl blue degradation in aqueous solution. In a typical photocatalytic measurement, 0.1 mg of c kana-Cu_3_(PO_4_)_2_ hybrid flowers was added into a 20 mL aqueous solution of the 5 mg L^−1^ methyl blue. The mixed solution was magnetically stirred for 30 minutes and left overnight in the dark to reach an equilibrium state before carrying out the photocatalytic reaction. The visible light source for the photocatalytic experiment is a 300 W air cooled Xenon lamp. At certain time points, 1.5 mL of mixed solution were taken out and centrifuged to remove the kana-Cu_3_(PO_4_)_2_ hybrid flowers. The photocatalytic performance for the MB removal was assessed by determine the absorbance at a wavelength of 653 nm. For the practical application, the kana-Cu_3_(PO_4_)_2_ hybrid flowers can be separated by centrifugation and use for the next cycle application.

## Supplementary information

Supplementary Information.
