# Supplementary material for: Self-assembled kanamycin antibiotic-inorganic microflowers and their application as a photocatalyst for the removal of organic dyes
Source: Sci Rep. 2020 Jan 13;10:154. doi: 10.1038/s41598-019-57044-z (PMC6957687; doi:10.1038/s41598-019-57044-z)
Supplement: Supplementary file 1 — Supplementary Information. [file 41598_2019_57044_MOESM1_ESM.docx]

**Electronic Supporting Information**

**Self-assembled kanamycin antibiotic-inorganic microflowers and their application as a photocatalyst for the removal of organic dyes**

Ratan W. Jadhav,^1,†^ Duong Duc La,^2,†^ Vishal G. More,^1^ Hoang Tung Vo,^3^ Duy Anh Nguyen^2^, Dai Lam Tran^4^, Sheshanath V. Bhosale^1,*^

^1^School of Chemical Sciences, Goa University, ​Taleigao Plateau, Goa 403 206, INDIA. ^2^Institute of Chemistry and Materials, Hanoi, Vietnam. ^3^Environmental Institute, Vietnam Maritime University, Haiphong city, Vietnam. ^4^Institute of Tropical Engineering, Vietnam Academy of Science and Technology, Hanoi, Vietnam. ^†^These authors contributed equally. *Corresponding email: svbhosale@unigoa.ac.in


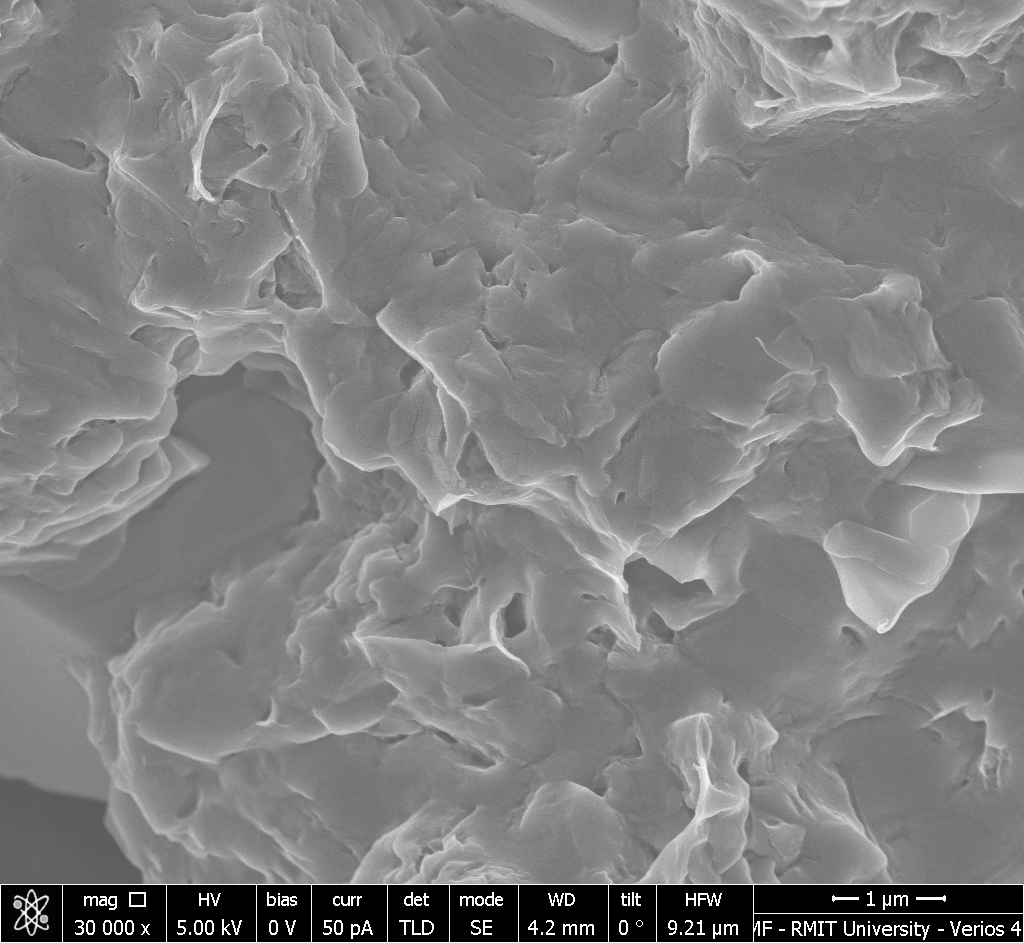


**Figure S1.** The high-resolution SEM image of Cu_3_(PO_4_)_2_­ crystals without addition of kanamycin

**
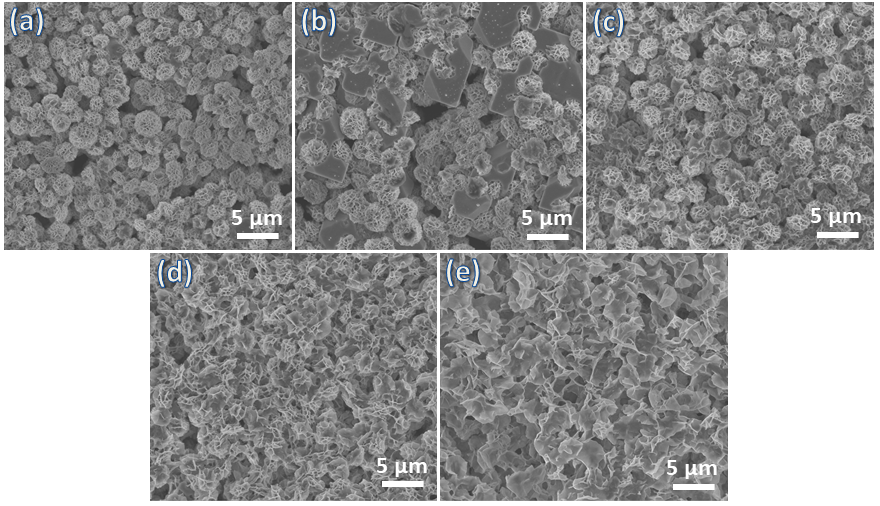
**

**Figure S2.** The SEM images of kanymicin-Cu_3_(PO_4_)_2_­ hybrid flowers formed with various kanamycin concentrations: a) 20 µl, b) 40 µl, c) 60 µl, d) 80 µl, 2) 100 µl.


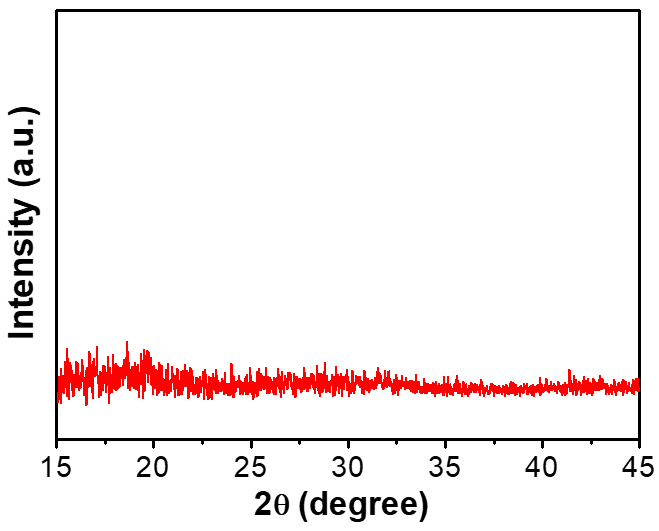


**Figure S3.** XRD pattern of monomeric Kanamycin molecules


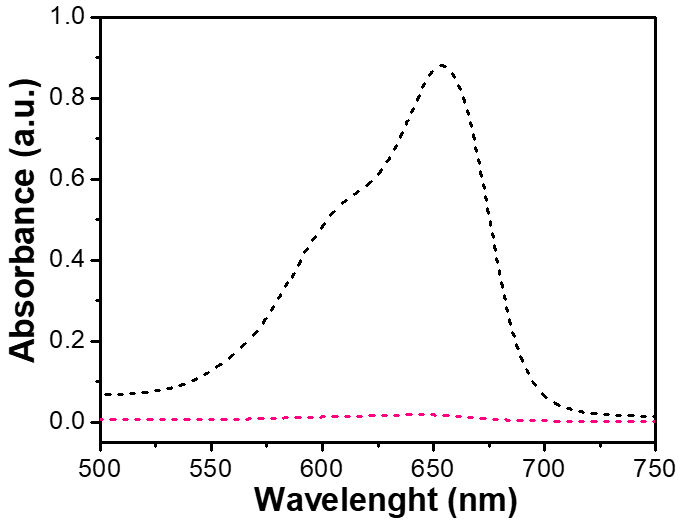


**Figure S4.** Absorption spectrum of MB solution (black line) and Kanamycin-Cu_3_(PO4)_2_ hybrid flowers in distilled water.


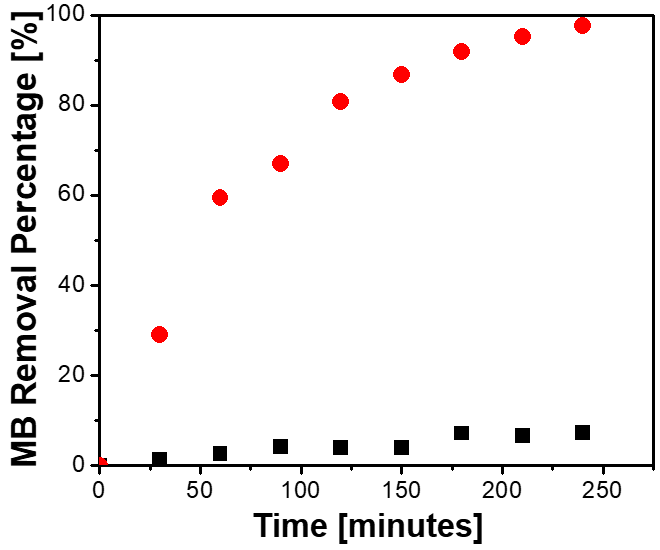


**Figure S5.** The methyl blue removal percentage using Kanymicin-Cu_3_(PO_4_)_2_­ hybrid flowers as photocatalyst.
